# Photocatalytic and antibacterial activities of Tl–Hg–I nanocomposites: sonochemical synthesis and characterization

**DOI:** 10.1039/d1ra03666a

**Published:** 2021-06-23

**Authors:** Elham Abkar, Mohammad Hassanpour, Omid Amiri, Mojgan Ghanbari, Masoud Salavati-Niasari

**Affiliations:** Institute of Nano Science and Nano Technology, University of Kashan Kashan 87317-51167 Iran salavati@kashanu.ac.ir +98 31 55913201 +98 31 55912383; Faculty of Chemistry, Razi University Kermanshah 6714414971 Iran; Department of Chemistry, College of Science, University of Raparin Rania Kurdistan Region Iraq

## Abstract

Efforts to find new and practical solutions to improve water quality and treatment of industrial effluents are ongoing. In this study, Tl_4_HgI_6_/HgI_2_ nanocomposites were synthesized by a rapid ultrasonic method to investigate their photocatalytic and antibacterial activity. Various synthesis conditions such as changes in the ratio of precursors, use of surfactants, and changes in the power and time of sonication to achieve particles with optimal size and morphology were performed. X-ray diffraction (XRD) and energy dispersive spectroscopy (EDS) analysis confirmed the purity and formation of the nanocomposite. Optimal nanoparticles in terms of size and morphology were selected by examining the images obtained from scanning electron microscopy (SEM) analysis. The nanocomposites obtained in the presence of PVP (polyvinylpyrrolidone) as a surfactant (sample no. 8) were selected as the optimal sample. Transmission electron microscopy (TEM), differential reflectance spectroscopy (DRS), Raman, N_2_ adsorption/desorption analyzes were performed for the optimal sample to evaluate the properties of nanocomposites. The band-gap for Tl_4_HgI_6_/HgI_2_ nanocomposites was calculated to be about 2.3 eV for HgI_2_ and 3.1 eV for Tl_4_HgI_6_. The optimal sample was used to evaluate the photocatalytic activity for decolorizing an aqueous solution of six different organic dyes. Finally, for rhodamine B, the decolorization was about 80%. Also, Tl_4_HgI_6_/HgI_2_ nanocomposite showed a significant inhibition zone in the antibacterial test. The maximum inhibition diameter of 50 mm was obtained against *Streptococcus pyogenes*. The results showed that Tl_4_HgI_6_/HgI_2_ nanocomposites have good potential for many industrial applications.

## Abstract 2

In this work, Tl_4_HgI_6_/HgI_2_ nanocomposites were synthesized by a rapid ultrasonic method. The photocatalytic properties of the nanocomposites were evaluated for the first time. Photocatalytic properties were performed for six industrial dyes.

## Graphical abstract

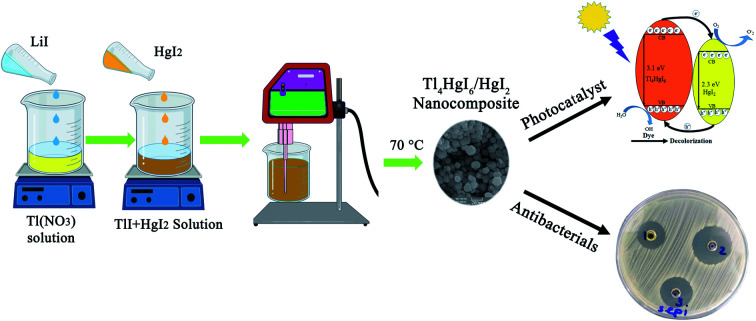

## 1. Introduction

In recent years, the use of nanomaterials has been evident in all industries.^1^ This ranges from the textile industry to pharmacy and water purification, and so on.^2–4^ Water as a source of life has always been a concern of researchers. Different methods for treating industrial effluents and drinking water have been proposed by researchers in recent years and are being developed. Due to the importance of water in human life, nanomaterials are also actively involved in improving and helping water resources to find new solutions in this direction.^5^ Nanomaterials have fruitful water treatment applications, such as membrane and filtration processes, precipitation processes, and especially photocatalytic processes.^6–8^

One of the most practical methods in improving the water quality of various pollutants, including dye contaminants in industrial effluents, is the photocatalytic process with nanomaterials under visible or UV light.^9^ In the photocatalysis process, several factors such as the concentration of contamination, the amount of photocatalyst, the type of light emitted, the pH of the environment, and some of the properties of the photocatalyst used in the efficiency of the process are effective.^10^ Among the required properties of a photocatalytic material are unique optical properties and the ability to produce electron holes after light irradiation.^11^ These unique properties exist in a class of materials called semiconductors. Semiconductors are of interest to researchers due to their excellent band-gap.^12^ Therefore, researchers have paid close attention to single crystal semiconductor materials with a wide band-gap.^13^ Among the attractive and encouraging items are materials with M_4_BX_6_ structure (M = Tl, In and B = Hg, Pb, Cd, and X = Cl, Br, I). Researchers in various fields have considered these three-halide semiconductors due to their electronic/optoelectronic properties.^14^

There are several ways to obtain nano-sized materials. Among the different methods for synthesis nanoparticles, co-precipitation, hydrothermal, microwave, and ultrasound can be mentioned in wet-chemical synthesis methods.^15^ The ultrasound is one of the fast and affordable methods.^16^ When the device is turned on, the released waves cause bubbles to form inside the solution. These bubbles, like small reactors, have a high temperature and pressure. The exploding of these bubbles and the release of energy, and the presence of these hot spots are considered one of the nanoparticle formation mechanisms.^17^ In addition to being fast and straightforward, this method is one of the methods considered by researchers due to its low energy consumption.^18^

In this project, Tl_4_HgI_6_/HgI_2_ nanocomposite was synthesized by the ultrasonic method. Different conditions were applied to achieve the appropriate morphology. This project aims to investigate less studied applications on these materials, including photocatalytic activity. Although some studies have shown the toxicity of some nanocomposite components (such as Hg and Tl), the nanocomposite activity against a group of bacteria and fungi was also investigated in this project. Thus, in some encounters with some microorganisms, it may be possible to use this nanocomposite.

## 2. Experimental

### 2.1. Materials and characterization

All reactants employed in this research were provided in high-grade quality. Mercury(ii) acetate (Hg(OAc)_2_), lithium iodide (LiI·2H_2_O), thallium(i) nitrate (TlNO_3_), polyvinyl pyrrolidone (PVP-25000), sodium salicylate (NaHSal), sodium dodecyl sulfate (SDS), ethylenediaminetetraacetic acid were purchased from Merck and utilized with no more refinement. The XRD of products was recorded by a Rigaku D-max CIII XRD using Ni-filtered Cu Kα radiation. SEM images were obtained on Philips XL-30ESEM, equipped with energy-dispersive X-ray spectroscopy. The EDS analysis with 20 kV accelerated voltage was done. An MPI Ultrasonic with the power of 1000 W, 20 kHz, from Switzerland (multi-wave ultrasound generator) by a transducer/converter was provided. A titanium oscillator was utilized for ultrasound radiation. The mixtures temperature *vs.* time was recorded for measuring the output power. This process was done in our previous work.^14,19^ The d*T*/d*t* evaluated plans from temperature (*T*) *vs.* time (*t*) data. Then the power of ultrasonic can estimate as follow:Power = (d*T*/d*t*) × *c*_p_ × *M*which *M* is the mass of water (solvent, kg), and *c*_p_ is the heat capacity of water (J kg^−1^ K^−1^). The calculated value from output power was estimated at 16.2 W, in the di-ionized water (for the input power of about 60 W).

### 2.2. Synthesis route

First, 0.2 g of thallium nitrate and 0.1 g of LiI·2H_2_O were dissolved in distilled water for 10 minutes on a magnetic stirrer, separately. Then two solutions were mixed. As a result, a yellow solution of thallium iodide was obtained. Separately, 0.087 g of Hg(OAc)_2_ was dissolved in distilled water on a magnetic stirrer for 10 minutes, and 0.075 g of LiI·2H_2_O was added to it. Finally, an orange mixture containing HgI_2_ was formed after a few minutes. The resulting solution was added to the first solution containing TlI. After 10 minutes of mixing, the resulting solution was sonicated. Different conditions were applied to obtain the optimal size and morphology, such as changing the ratio of precursors, adding surfactants, and changing the time of use and ultrasonic power, which are detailed in Table 1. All reaction steps were performed at ambient temperature. The resultant precipitates were filtered, washed using distilled water, and dried in an oven at 70 °C.

**Table 1:** Different reaction conditions for the synthesis of Tl_4_HgI_6_/HgI_2_ nanocomposite

Sample no.	TlI : HgI_2_ ratio	Surfactant	Time of sonication (minutes)	Power of sonication (W)	Grain size (nm)
1	1	—	20	60	33
2	2	—	20	60	30
3	3	—	20	60	26
4	0.5	—	20	60	31
5	2	SDS	20	60	27
6	2	NaHSal	20	60	26
7	2	EDTA	20	60	35
8	2	PVP	20	60	33
9	2	PVP	20	40	25
10	2	PVP	20	80	23
11	2	PVP	10	60	31
12	2	PVP	30	60	30

### 2.3. Photocatalysis process

Tl_4_HgI_6_/HgI_2_ nanocomposite photocatalytic test was performed under UV light for six organic dyes. The photocatalysis procedure was performed as follows: 0.03 g of the nanocomposite was dispersed in 50 ml of a 5 ppm dye solution in a quartz tube at ambient temperature. The resulting suspension was aerated in a dark environment for 20 minutes before applying UV light to achieve absorption and excretion equilibrium. After aeration, the resulting solution was exposed to UV light. The solution was sampled for 90 minutes at specified times. The collected samples were centrifuged to separate the nanocomposite from the dye solution. Then the samples were analyzed by UV-vis spectrophotometer. The efficiency of Tl_4_HgI_6_/HgI_2_ nanocomposite in photocatalytic process in decolorization is calculated as follows:^20^% decolorization= ((*A*_t_ − *A*_0_)/*A*_0_) × 100;where *A*_0_ and *A*_t_ is the amount of dye absorption before and after exposure to light.

### 2.4. Antibacterial test

The conventional well diffusion (WD) method was used to evaluate the antibacterial activity of the Tl_4_HgI_6_/HgI_2_ nanocomposite. The nutrient agar medium was used in order to support the growth of the bacteria. For the preparation of 1000 ml nutrient agar medium, primarily, 28 g nutrient powder was dissolved in 1000 ml distilled water. This medium was autoclaved at 121 °C under 15 lbs pressure for 20 minutes. The following microbial strains were used in order to investigate the activity of the Tl_4_HgI_6_/HgI_2_ nanocomposite as; *Aspergillus niger* (ATCC 9029), *Aspergillus brasiliensis* (ATCC 16404), *Bacillus subtilis* (ATCC 6633), *Candida albicans* (ATCC 10231), *Escherichia coli* (ATCC 25922), *Klebsiella pneumonia* (ATCC 10031), *Pseudomonas aeruginosa* (ATCC 27853), *Salmonella paratyphi-A* serotype (ATCC 5702), *Shigella dysenteriae* (PTCC 1188), *Staphylococcus aureus* (ATCC 29737), *Staphylococcus epidermidis* (CIP 81.55), *Streptococcus pyogenes* (ATCC 19615). The freshly grown liquid culture of the bacteria was dispersed in agar plates. 300 mg ml^−1^ of the Tl_4_HgI_6_/HgI_2_ nanocomposites were injected into the wells and kept in an incubator at 37 °C for 24 h to examine the inhibition effect of the nanocomposites on the freshly grown cultures. After complete incubation, the zone of inhibition was measured. Finally, the bacterial strains and yeast sensitivity to Tl_4_HgI_6_/HgI_2_ nanocomposites were investigated for their minimal inhibition concentration (MIC) values using the micro-well dilution assay method.^21^

## 3. Result and discussion

**Fig. 1:**
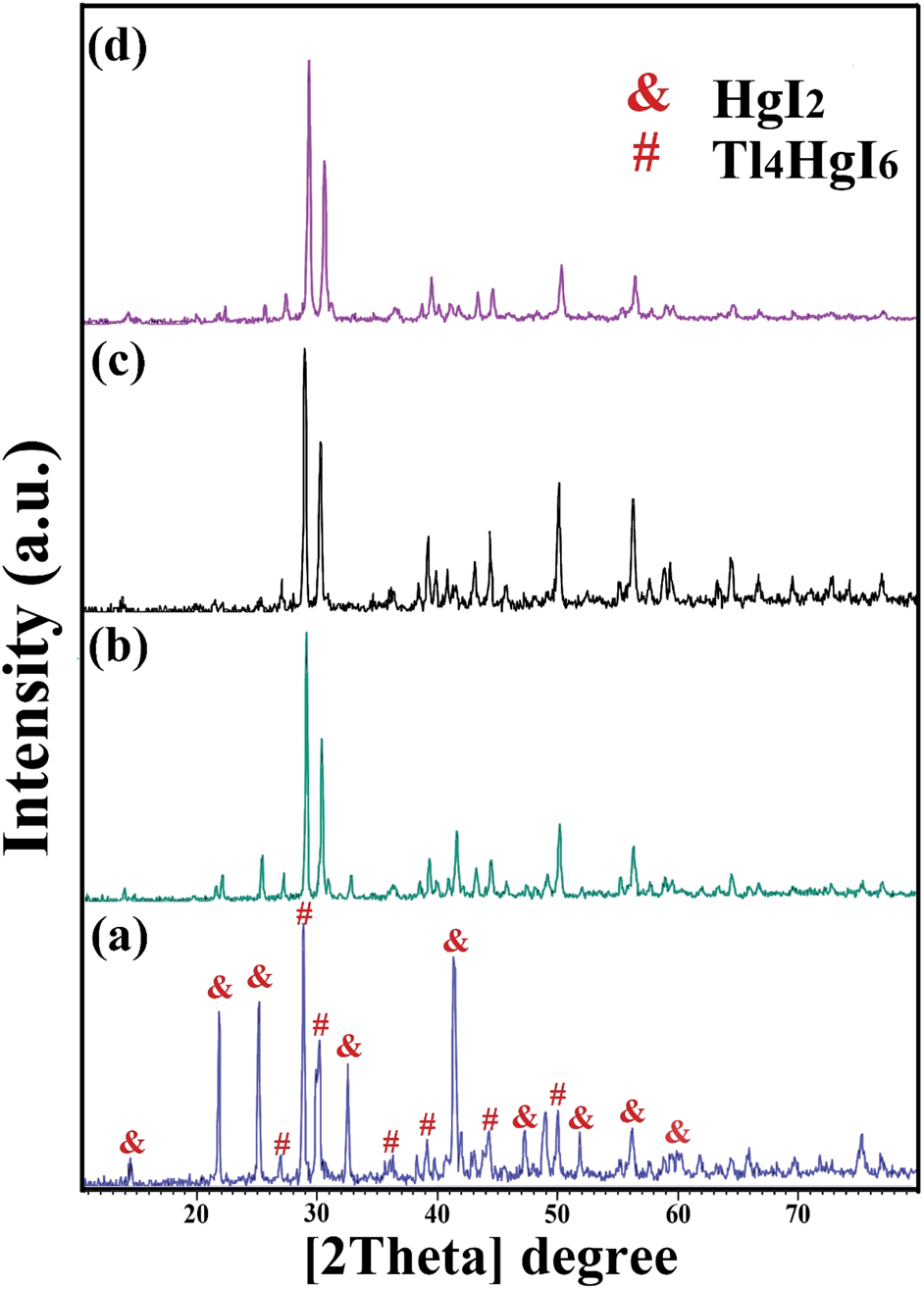
XRD patterns obtained from Tl_4_HgI_6_/HgI_2_ nanocomposite in different ratios of Tl to HgI_2_ precursors; (a) 1 : 1, (b) 2 : 1, (c) 3 : 1, and (d) 1 : 2.

**Fig. 2:**
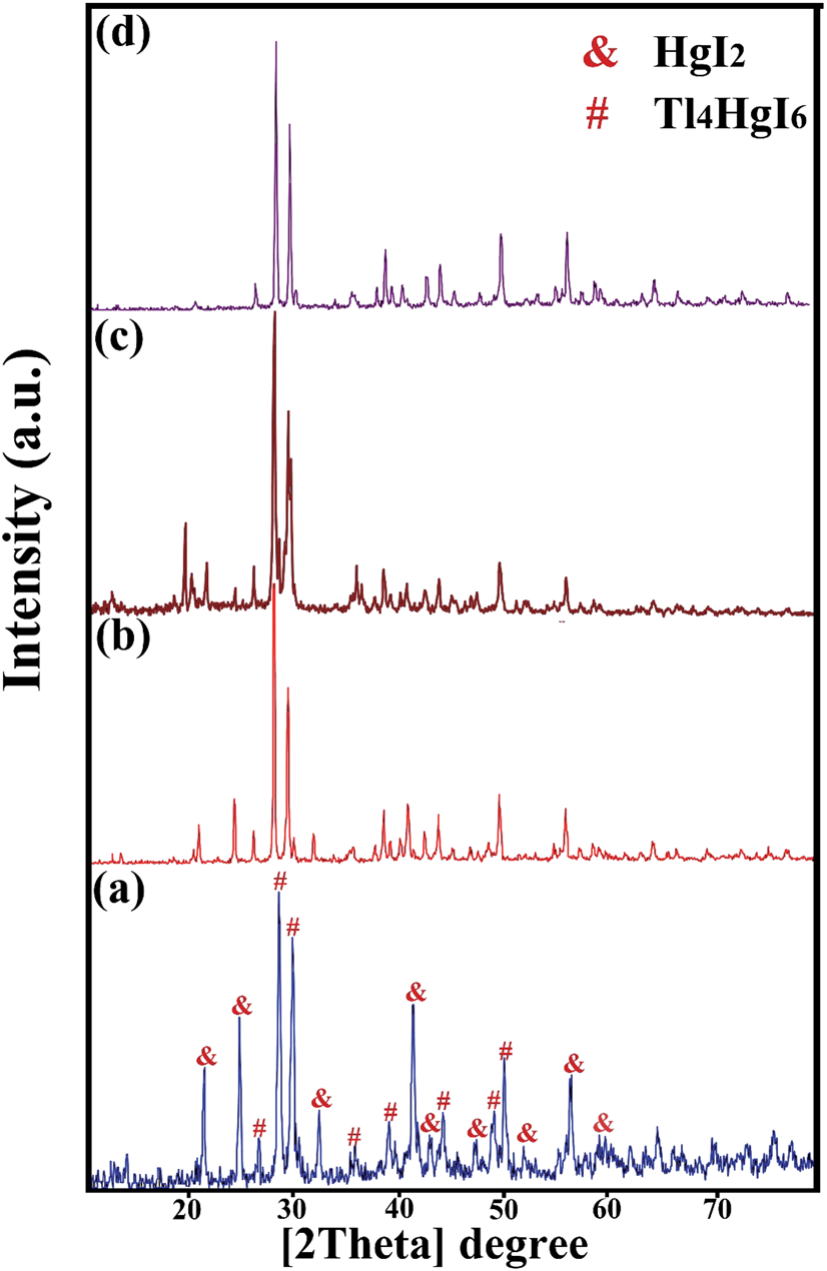
XRD patterns obtained from Tl_4_HgI_6_/HgI_2_ nanocomposite in different surfactant; (a) SDS, (b) NaHSal, (c) EDTA, and (d) PVP.

**Fig. 3:**
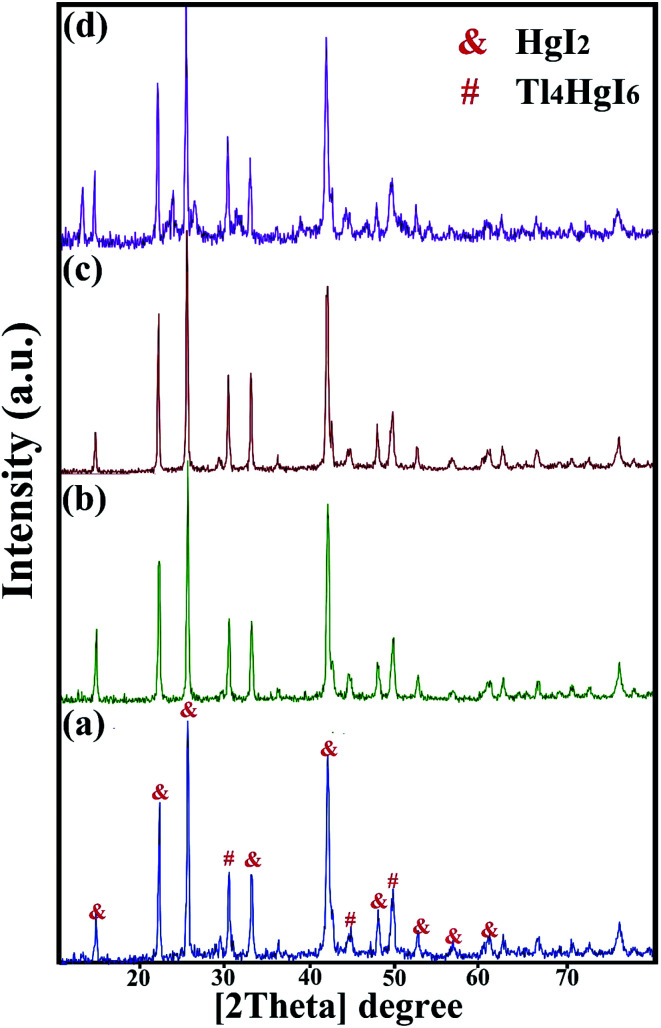
XRD patterns obtained from Tl_4_HgI_6_/HgI_2_ nanocomposite in a different time and power of sonication; (a) 20 minutes, 40 watt, (b) 20 minutes, 80 watt, (c) 10 minutes, 60 watt, and (d) 30 minutes, 60 watt.

XRD analysis was performed on all samples to investigate the crystal lattice and nanocomposite formation. All samples confirmed the formation of the Tl_4_HgI_6_/HgI_2_ nanocomposite. As shown in Fig. 1, 2, and 3, the XRD pattern confirms the presence of both the tetragonal phase of Tl_4_HgI_6_ (JCPDS no. 16-0212, space group *P*4_2_*bc*) and HgI_2_ (JCPDS no. 4-0455). Crystalline sizes were calculated from the Scherrer equation, *D*_c_ = *Kλ*/*β* cos *θ*.^22^ The crystalline size for nanocomposites for each XRD pattern was calculated separately and listed in Table 1. The average crystallite size of the synthesized nanocomposite was calculated at about 29 nm.

Fig. 1 (a–d) shows the XRD pattern of synthesized nanocomposites in different ratios of precursors (sample no. 1–4, respectively). By changing the ratio of substances and reducing the amount of HgI_2_, the intensity of the prominent peaks related to HgI_2_ in the 2*θ*: 22, 25, 33, 41, and 56 were significantly reduced. Different results were obtained by adding surfactant in the synthesis steps. As shown in Fig. 2(a–d) (sample no. 5–8, respectively), the characteristic peaks of HgI_2_ reappeared with appropriate intensity by the addition of SDS. The intensity of these peaks decreased when NaHSal was added, and when utilizing EDTA, a new peak appeared at about 2*θ*:20 degrees. When PVP was used as a surfactant, the HgI_2_ index peaks disappeared, and only peaks in the range of 41 and 56 degrees with low intensity can be seen in the pattern.

Next, to further explore the instrumental factors, including time and power, two different times and power were tested to evaluate the effect of application time and device power. Fig. 3 (a–d) showed the XRD pattern of the nanocomposite when the device parameters changed (sample no. 9–12, respectively). In all four patterns, the characteristic peaks of HgI_2_ increased significantly by changing the time and power of the device.

In the synthesis of nanoparticles by ultrasonic method, several general mechanisms are proposed: forming bubbles and hot spots, radical production, and the formation of vesicles.^18,23,24^ However, in the synthesis of Tl_4_HgI_6_/HgI_2_ nanocomposites, what is most likely is the mechanism of the formation of bubbles and hot spots in the synthesis medium. The formation and bursting of these bubbles increased the temperature, which promotes the reaction to the product. The progress of the reaction is summarized below:

1 2Tl(NO_3_) + LiI_2_ → 2TlI

2 Hg(OAc)_2_ + LiI_2_ → HgI_2_

3
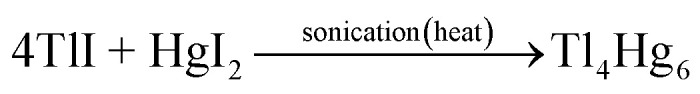


**Fig. 4:**
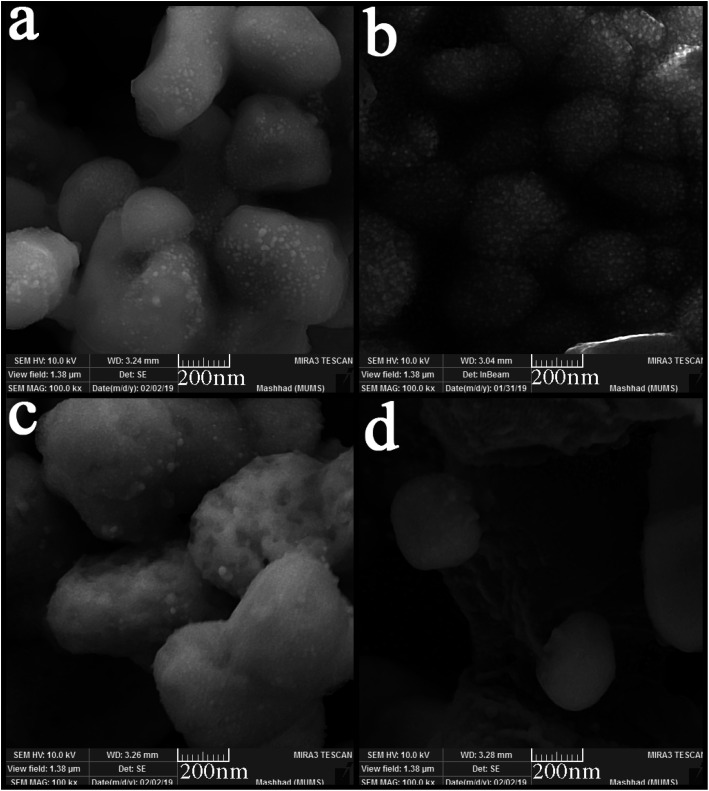
SEM images of synthesized nanocomposites using different ratios of Tl to HgI_2_ precursors; (a) 1 : 1, (b) 2 : 1, (c) 3 : 1, and (d) 1 : 2.

**Fig. 5:**
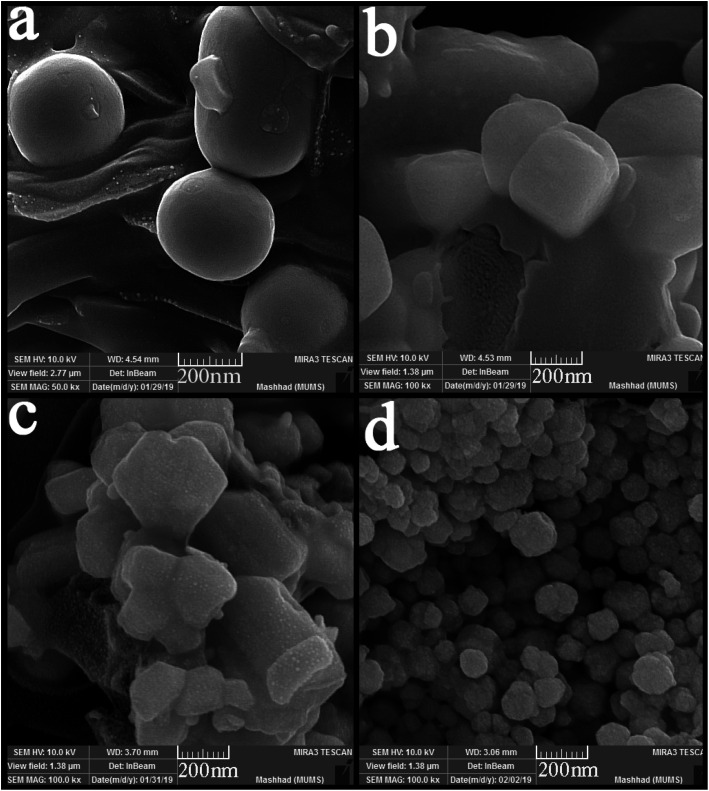
SEM images of synthesized nanocomposites using different surfactant; (a) SDS, (b) NaHSal, (c) EDTA, and (d) PVP.

SEM images were applied to study the morphology of the synthesized nanocomposites. Fig. 4 (a–d) shows the SEM images of synthesized nanocomposites using different ratios of precursors. The particles stuck together in almost all the proportions used and formed large balls. However, Fig. 4(b) shows small agglomerated particles when the precursors were used in a 2 : 1 ratio. Different surfactants were used to obtain particles with a suitable size and morphology. Fig. 5(a–d) shows the SEM images of nanocomposites synthesized in the presence of surfactants. The obtained images show that only when PVP was used as a surfactant (Fig. 5(d)), the particle size changed, and the particle size became more petite than the other. When other surfactants such as SDS, EDTA, and NaHSal were used, micrometer-sized bullet-shaped particles were obtained. Due to the appropriate size obtained when PVP was used as a surfactant, it was selected as a suitable surfactant in continuing studies.

**Fig. 6:**
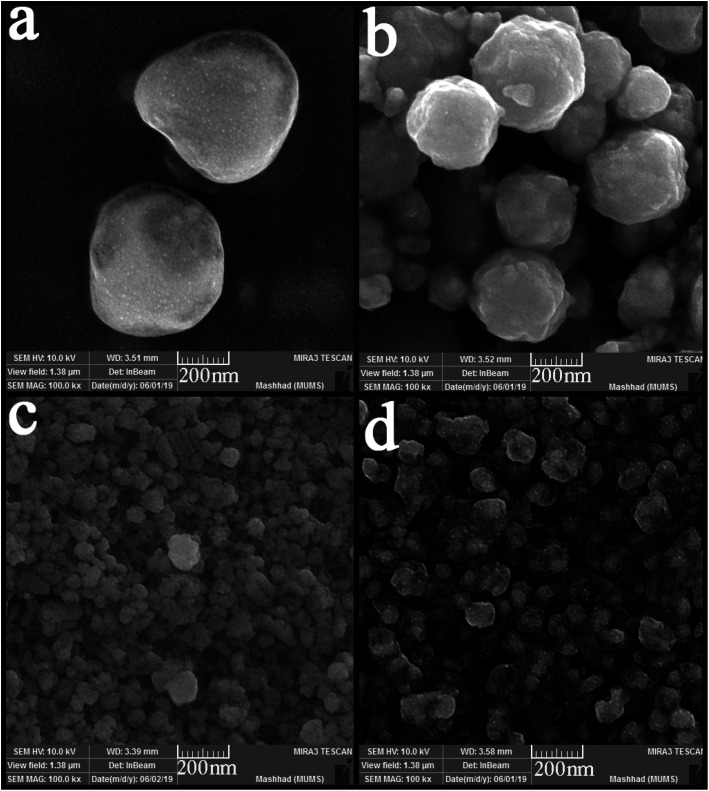
SEM images of synthesized nanocomposites using different time and power of sonication; (a) 20 minutes, 40 watt, (b) 20 minutes, 80 watt, (c) 10 minutes, 60 watt, and (d) 30 minutes, 60 watt.

The time and power of sonication as influencing factors in particle size and morphology were investigated in the nanocomposite synthesis process. Two different times and powers (10 and 30 minutes, 40 and 80 watts, respectively) were applied in this study. As can be seen from Fig. 6 (a–d), the change in power did not positively affect particle size or a notable change in particle morphology. The spherical particles with micrometer sizes were obtained as in the previous samples (Fig. 6(a and b)). However, the particle size changed significantly with the change in the sonication time. Smaller and monodisperse particles were formed when the time of sonication was 10 minutes. However, some agglomeration is observed because of the high activity of nanoparticles. In contrast, by increasing the time to 30 minutes, the particles agglomerated and formed masses because of the temperature increasing and interaction of nanoparticles.

**Fig. 7:**
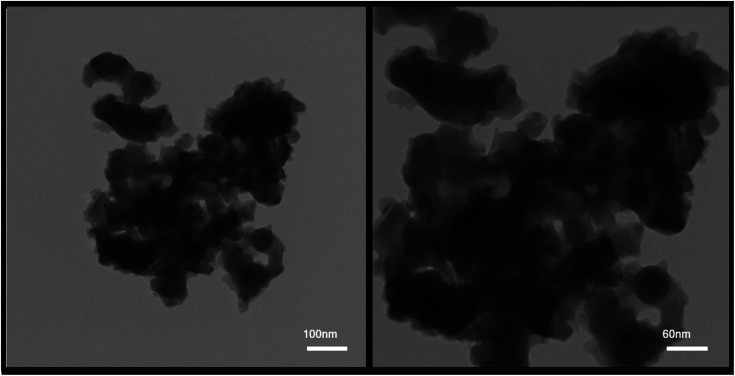
TEM images from the sample prepared in the presence of PVP as the optimal sample (sample no. 8).

According to the XRD analysis and review of images obtained from SEM analysis, sample no. 8 was selected as the optimal sample in terms of purity and appropriate size. The optimal sample was used for the rest of the supplementary analyzes in the continuation of the project. Fig. 7 shows the TEM images from the sample prepared in the presence of PVP as the optimal sample (sample no. 8) in two magnifications. As can be seen, the nanoparticles are adhered together and agglomerated. According to the images, this event increased the particle size, which was predictable in the synthesis of nanoparticles due to their high reactivity.

**Fig. 8:**
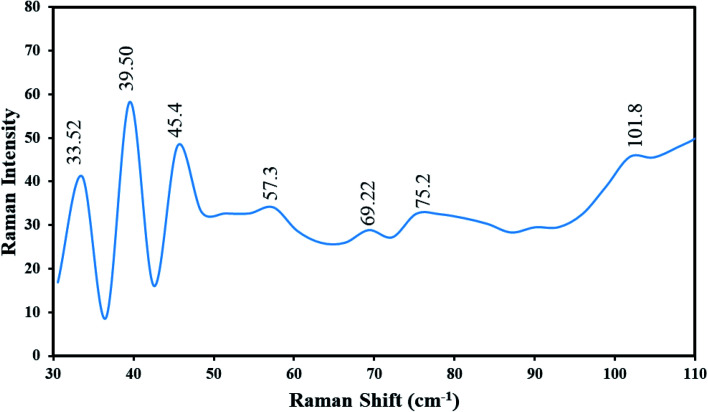
Raman spectrum of Tl_4_HgI_6_/HgI_2_ nanocomposite (sample no. 8) at ambient temperature (25 °C).

Fig. 8 demonstrates the Raman spectrum of Tl_4_HgI_6_/HgI_2_ nanocomposites (sample no. 8) at ambient temperature (25 °C). The distinct absorptions are symbolized in the range of 30–110 cm^−1^. Thus, seven lines were found in the spectrum of Tl_4_HgI_6_/HgI_2_ nanocomposites, which were located at 33.52, 39.50, 45.47, 57.39, 69.29, 75.22, and 101.85 cm^−1^. All seven lines are following Tl_4_HgI_6_/HgI_2_.^25^ Also, these vibrational lines can be allocated by regarding the Tl_4_HgI_6_ containing the vibrational states of TlI and HgI_2_. The peak at 57.39 was ascribed to the Tl–I, and the peak at 69.29 cm^−1^ is attributed to the symmetric stretching of the Tl–I.^14^ The Raman bands at 101.85, 45.47, and 33.52 cm^−1^ are allocated to HgI_2_.^26^ The Raman shift of Tl_4_HgI_6_/HgI_2_ nanocomposites was agreed to the Raman shift rates of Tl_4_HgI_6_ mentioned in the literature. The results propose a blue shift in the Raman spectrum owing to the captivity of phonon and stress influence, which is caused by reducing the particle size.^27,28^

**Fig. 9:**
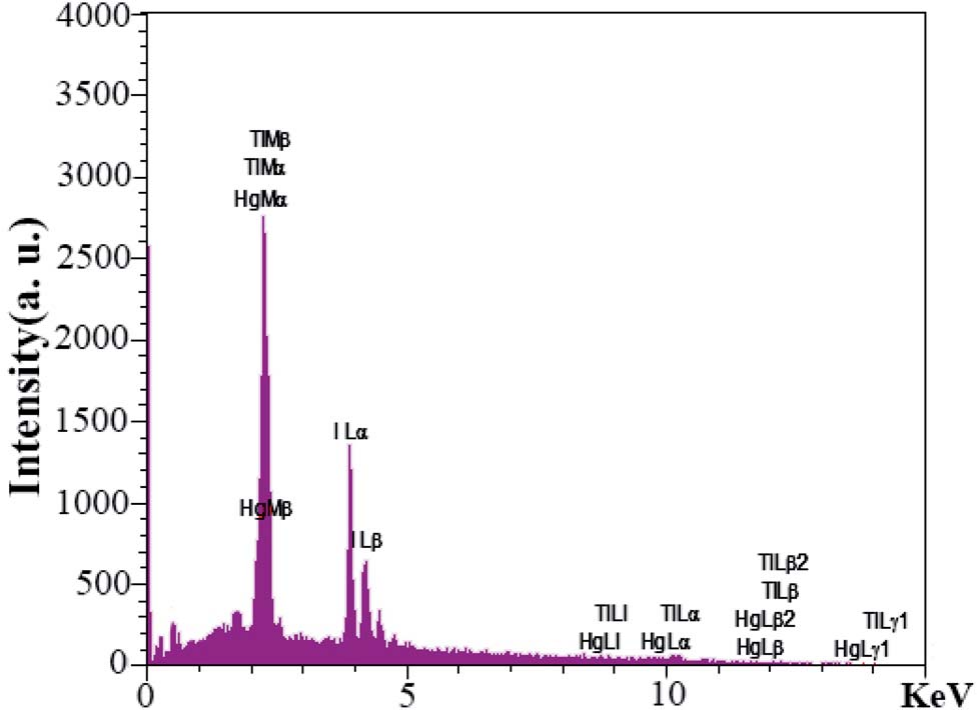
EDS analyses from sample no. 8 as the optimal sample.

EDS analysis was used to investigate the presence of elements were used in the synthesis of nanocomposites. The EDS analysis output in Fig. 9 confirmed Tl, Hg, and I elements in the nanocomposites. Also, it showed that no element was present as an impurity in the nanocomposites.

**Fig. 10:**
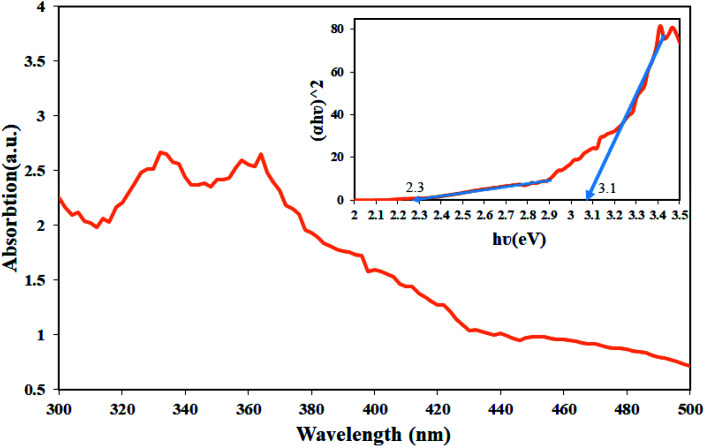
DRS analyses from sample no. 8 as the optimal sample and inset resulting graph to estimate the band-gap of Tl_4_HgI_6_/HgI_2_ nanocomposite.

One of the main parameters in detecting semiconductor materials is the band-gap value. DRS analysis was used to investigate the energy band of the synthesized nanocomposite, which was performed in the wavelength range of 300 to 500 nm for Tl_4_HgI_6_/HgI_2_ nanocomposites. The resulting spectrum is shown in Fig. 10. Tauc's equation was used to obtain the band-gap.^29^ The results obtained from these calculations in the form of diagrams (*αhν*^2^) in terms of (*hν*) inside the DRS diagram are quite clear. The estimated band-gap for this nanocomposite was about 2.3 eV for HgI_2_ and 3.1 eV for Tl_4_HgI_6_. Values of 2.1 and 2.5 eV have been reported for HgI_2_ and Tl_4_HgI_6_ particles in the literature.^30,31^ The difference between obtained and the reported values can be attributed to particle size change. As the particle size decreases, the band-gap increases.^32^

**Fig. 11:**
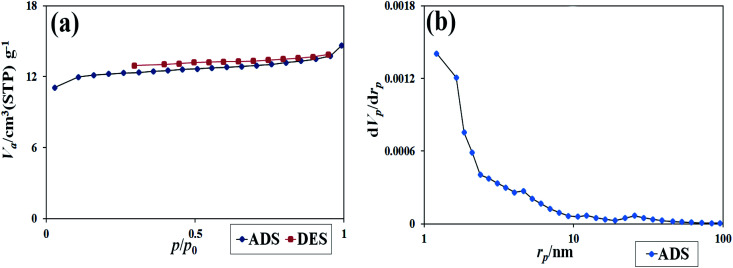
(a) The isotherm obtained from the study of N_2_ adsorption/desorption on Tl_4_HgI_6_/HgI_2_ nanocomposite, (b) the graph obtained from BJH calculations of sample no. 8.

Absorption and desorption of N_2_ at 77 K were used to evaluate the pores and specific surface area of the synthesized nanocomposites. As is clear from Fig. 11, the type III isotherm with a type H3 hysteresis loop for the nanocomposites was obtained.^22^ The data were obtained using BET and BJH calculations of specific surface area, the total pore volume, and the mean pore diameters. The specific surface area was 46.47 (m^2^ g^−1^), the total pore volume was 10.67 (cm^3^ g^−1^), and the mean pore diameter was 1.21 nm. The results showed that a suitable surface area of the nanocomposite could be provided. This surface area contributes significantly to dye absorption and the number of active sites in the photocatalysis process.^33,34^

**Fig. 12:**
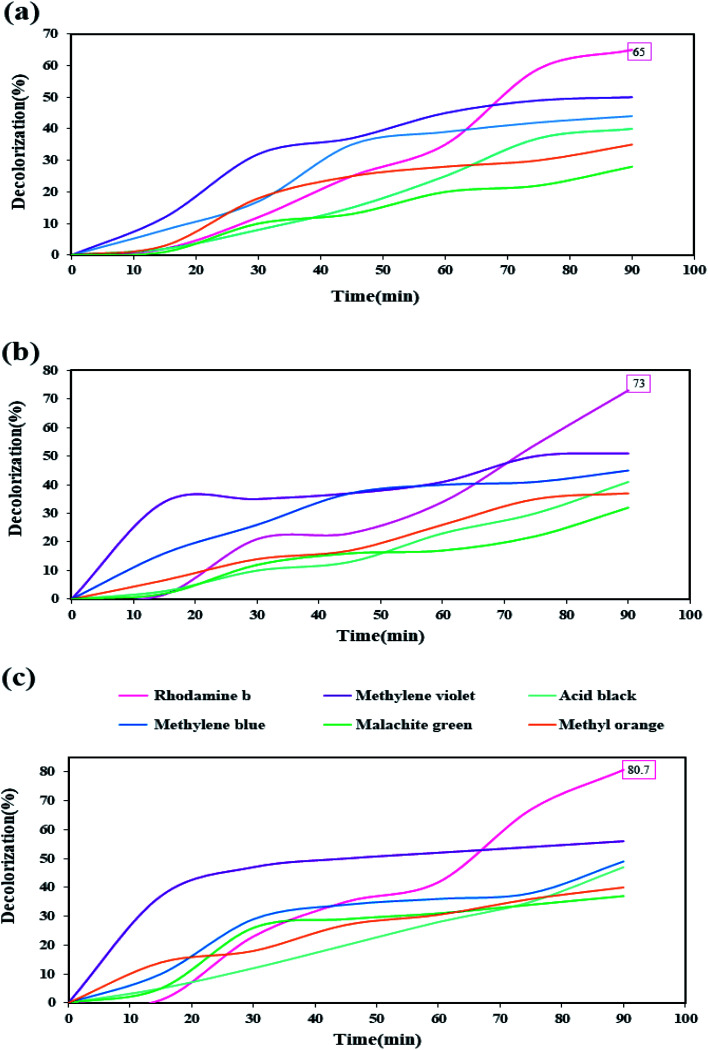
Photocatalysis test results for six dyes in the presence of different amounts of Tl_4_HgI_6_/HgI_2_ nanocomposite (a) 0.03 gr, (b) 0.05 gr, and (c) 0.07 gr.

After performing diagnostic analyses and confirming the formation and purity of the nanocomposite, and examining some properties of the synthesized nanocomposite due to the obtained band-gap, this nanocomposite was used to evaluate its decolorization ability against organic dyes under UV light in the photocatalysis process. Six organic dyes include: rhodamine b, methylene violet, acid black, methylene blue, malachite green, and methyl orange, were used in this photocatalytic test. The photocatalysis process was performed for 90 minutes under UV light. Photocatalysis testing was performed in three values of 0.03, 0.05, and 0.07 gr of the nanocomposite (Fig. 12 (a–c), respectively). Among these six dyes, rhodamine was the only dye that showed a decolorization of over 60%. With increasing the amount of nanocomposite was used as the photocatalyst, the percentage of decolorization also increased in different dyes. When 0.07 gr of the nanocomposite was used, the highest decolorization percentage was obtained for rhodamine b, which was about 80%.

The increase in the percentage of decolorization with increasing the amount of catalyst can be due to increase the active sites and further increase the absorption of dye on the catalyst surface.^35,36^ Reviewing various articles showed that this nanocomposite might be a type-II heterojunction.^37–39^ Therefore, it is also possible to imagine the photocatalytic process when HgI_2_ and Tl_4_HgI_6_ nanoparticles are together, as fallow: after UV light irradiates the Tl_4_HgI_6_ particles, which has a higher band-gap, the generated electrons are transferred to the conduction band of the HgI_2_. This electron–hole circulation increases the recombination times, increasing the number of free radicals produced. An increase in the number of free radicals increases the amount of decolorization.^40–42^

**Fig. 13:**
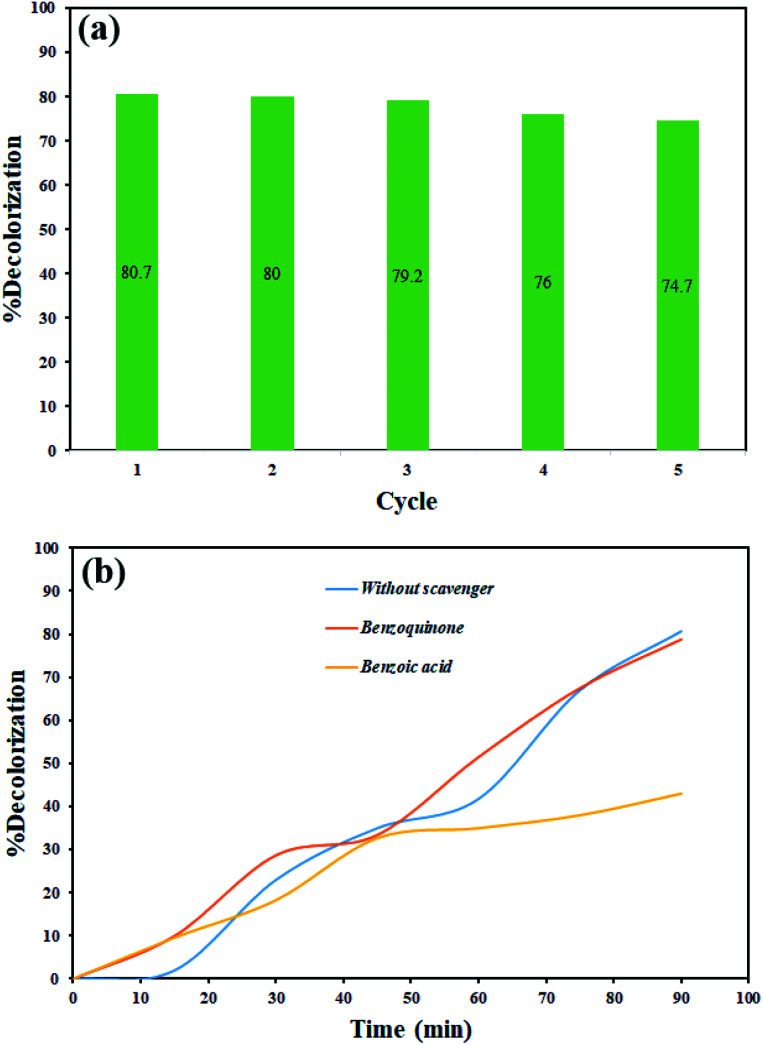
(a) Evaluation of the stability of photocatalytic activity of Tl_4_HgI_6_/HgI_2_ nanocomposite on rhodamine b decolorization, (b) photocatalytic activity of Tl_4_HgI_6_/HgI_2_ nanocomposite in the presence of scavengers.

The photocatalysis process was repeated five times for rhodamine b to evaluate the stability of the photocatalytic activity of the synthesized nanocomposite. As shown in Fig. 13(a), the photocatalytic activity did not show a significant change, although there was a slight decrease in decolorization percentage.

The scavengers were used to evaluate which radical species are effective in dye decolorization. Various materials are applied to trap free radicals.^43,44^ 1,4-Benzoquinone and benzoic acid were used to remove superoxide, and hydroxide radicals, respectively. Therefore, photocatalysis testing was done in the presence of these materials for rhodamine b under the same conditions. As shown in Fig. 13(b), the results showed that hydroxide radical as a free radical produced in the photocatalytic process plays a significant role in the decolorization of dye contamination in the presence of Tl_4_HgI_6_/HgI_2_ nanocomposite. According to the results, the mechanism of the photocatalytic process can be considered as follows:4
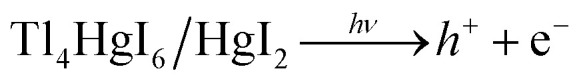

5 h^+^ + H_2_O → H^+^ + OH

6 h^+^ + OH^−^ → OH

7 e^−^ + O_2_ → O_2_^−^

8 OH + dye contamination → decolorization product

After studying the photocatalytic properties of the synthesized nanocomposite, this nanocomposite was used against several bacteria and fungi. The results of antibacterial testing in the well-diffusion method were very significant and exciting. Comparing the test results with the famous antibiotics rifampin and gentamicin, the obtained inhibitor zone diameter showed that the bacteria are susceptible to this nanocomposite. The highest inhibitor zone diameter was obtained for *Staphylococcus aureus* and *Streptococcus pyogenes* about 40 and 50 mm. The results of antibacterial activity and control test of common antibiotics are given in Tables 2 and 3, respectively. These extraordinary results were predictable due to the presence of HgI_2_ in the nanocomposite and its antibacterial properties, which have been confirmed in other works.^45,46^ The mechanism of ROS production in nano-sized materials is also one of the most likely mechanisms inhibiting bacterial growth.^47^

**Table 2:** Results of antibacterial test of Tl_4_HgI_6_/HgI_2_ nanocomposite^a^

Test microorganism	DZ	MIC	MBC	Test microorganism	DZ	MIC	MBC
*Aspergillus niger*	23	125	125	*Pseudomonas aeruginosa*	18	<3.91	125
*Aspergillus brasiliensis*	22	250	250	*Salmonella paratyphi-A* serotype	31	7.81	62.50
*Bacillus subtilis*	27	7.81	62.50	*Shigella dysenteriae*	31	7.81	31.25
*Candida albicans*	23	<3.91	>3.91	*Staphylococcus aureus*	40	31.25	125
*Escherichia coli*	28	<3.91	<3.91	*Staphylococcus epidermidis*	18	15.63	62.50
*Klebsiella pneumonia*	32	7.81	62.50	*Streptococcus pyogenes*	50	>3.91	>3.91

a DZ: inhibition zone in diameter (mm), MIC: minimal inhibition concentration as mg mL^−1^, MBC: minimum bactericidal concentration as mg mL^−1^.

**Table 3:** Results from the study of conventional antibacterial drugs for various bacteria^a^

Antibiotics	Rifampin		Gentamicin		Antibiotics	Rifampin		Gentamicin	
Microorganism	DZ	MIC	DZ	MIC	Microorganism	DZ	MIC	DZ	MIC
*Aspergillus Niger*	NA	NA	NA	NA	*Pseudomonas aeruginosa*	—	31.25	20	7.81
*Aspergillus brasiliensis*	NA	NA	NA	NA	*Salmonella paratyphi-A* serotype	8	15.63	18	3.90
*Bacillus subtilis*	19	31.25	30	3.9	*Shigella dysenteriae*	9	15.36	17	3.90
*Candida albicans*	NA	NA	NA	NA	*Staphylococcus aureus*	21	31.25	27	1.95
*Escherichia coli*	11	3.90	20	3.90	*Staphylococcus epidermidis*	44	1.95	39	1.95
*Klebsiella pneumonia*	8	15.63	17	3.90	*Streptococcus pyogenes*	21	0.975	32	0.975

a DZ: inhibition zone in diameter (mm), MIC: minimal inhibition concentration as mg mL^−1^, NA: no activity.

Comparison of the obtained results with the results of previous work in which only Tl_4_HgI_6_ was synthesized,^48^ showed that the presence of HgI_2_ beside Tl_4_HgI_6_ and preparing the nanocomposite improved the antibacterial and photocatalytic activities of Tl_4_HgI_6_. For example, the inhabitation zone of microorganisms in the current study (Tl_4_HgI_6_/HgI_2_ nanocomposites) is much larger than the previous study (Tl_4_HgI_6_ nanostructures). Tl_4_HgI_6_/HgI_2_ nanocomposites degraded rhodamine b about 80% under UV light, Tl_4_HgI_6_ degraded 76% under the same condition. These outcomes revealed that the presence of HgI_2_ enhanced photocatalytic and antibacterial performances.

## 4. Conclusion

In summary, the Tl_4_HgI_6_/HgI_2_ nanocomposite was synthesized by the ultrasonic method. Various conditions were applied in the synthesis method, including the addition of surfactant, changing the ratio of precursors, and changing the conditions of the device to obtain nanocomposites with appropriate size and morphology. After reviewing the results obtained from XRD and SEM analyzes, the synthesized sample was selected as the optimal sample in the presence of PVP with a time of 20 minutes and a power of 60 watts. Numerous analyzes were performed to evaluate the properties of nanocomposites on the optimal sample. The DRS analysis results and band-gap calculation provided suitable optical properties of this nanocomposite for the photocatalytic process. The photocatalysis process was performed for six organic dyes as well as in three different nanocomposite quantities. The photocatalytic activity of Tl_4_HgI_6_/HgI_2_ nanocomposite for rhodamine b in the highest amount of nanocomposite (0.07 gr) showed the highest percentage of decolorization (about 80%). This nanocomposite was also used to study its antibacterial properties against several bacteria and fungi using the well-diffusion method. Extraordinary results were obtained in the study of antibacterial properties. The inhibition zone diameter for *Staphylococcus aureus* was about 40 mm and for *Streptococcus pyogenes* was about 50 mm, which was significantly different from the halos obtained from conventional antibiotics. Generally, the results show the potential of this nanocomposite to investigate other applications, but the toxicity of this nanocomposite and its components in all processes should be investigated.

## Conflicts of interest

The authors declare that there are no conflicts of interest regarding the publication of this manuscript.

## Supplementary Material

## References

[cit1] Singh N. A. (2017). Environ. Chem. Lett..

[cit2] Shabbir M. and Kaushik M., in Handbook of Nanomaterials for Manufacturing Applications, Elsevier, 2020, pp. 249–263

[cit3] Mozafari M. R. , in Nanomaterials and nanosystems for biomedical applications, Springer Science & Business Media, 2007

[cit4] Khan S. T., Malik A. (2019). J. Hazard. Mater..

[cit5] Das R., Vecitis C. D., Schulze A., Cao B., Ismail A. F., Lu X., Chen J., Ramakrishna S. (2017). Chem. Soc. Rev..

[cit6] Wang D. (2019). Cellulose.

[cit7] Fan H.-L., Zhou S.-F., Jiao W.-Z., Qi G.-S., Liu Y.-Z. (2017). Carbohydr. Polym..

[cit8] Ganguly P., Harb M., Cao Z., Cavallo L., Breen A., Dervin S., Dionysiou D. D., Pillai S. C. (2019). ACS Energy Lett..

[cit9] Tahir M., Ali S., Rizwan M. (2019). Int. J. Environ. Sci. Technol..

[cit10] Kumar A., Pandey G. (2017). Mater. Sci. Eng. Int. J..

[cit11] Reddy C. V., Babu B., Shim J. (2018). J. Phys. Chem. Solids.

[cit12] Ansari F., Sobhani A., Salavati-Niasari M. (2018). J. Colloid Interface Sci..

[cit13] Kim M., Seo J.-H., Singisetti U., Ma Z. (2017). J. Mater. Chem. C.

[cit14] Ghanbari M., Salavati-Niasari M. (2018). Inorg. Chem..

[cit15] Nikam A., Prasad B., Kulkarni A. (2018). CrystEngComm.

[cit16] Mohanty P., Mahapatra R., Padhi P., Ramana C. V., Mishra D. K. (2020). Nano-Struct. Nano-Objects.

[cit17] Ashokkumar M. , in Ultrasonic synthesis of functional materials, Springer, 2016, pp. 17–40

[cit18] Hassanpour M., Tafreshi S. A. H., Amiri O., Hamadanian M., Salavati-Niasari M. (2020). Chemosphere.

[cit19] Gholamrezaei S., Ghanbari M., Amiri O., Salavati-Niasari M., Foong L. K. (2020). Ultrason. Sonochem..

[cit20] Dara M., Hassanpour M., Alshamsi H. A., Baladi M., Salavati-Niasari M. (2021). RSC Adv..

[cit21] Hassanpour M., Salavati-Niasari M., Tafreshi S. A. H., Safardoust-Hojaghan H., Hassanpour F. (2019). J. Alloys Compd..

[cit22] Hassanpour M., Salavati-Niasari M., Safardoust-Hojaghan H. (2020). Environ. Sci. Pollut. Res..

[cit23] Ghiyasiyan-Arani M., Salavati-Niasari M., Naseh S. (2017). Ultrason. Sonochem..

[cit24] Yousefi S. R., Amiri O., Salavati-Niasari M. (2019). Ultrason. Sonochem..

[cit25] Yukhymchuk V. O., Dzhagan V. M., Mazur N. V., Parasyuk O. V., Khyzhun O. Y., Luzhnyi I. V., Yaremko A. M., Valakh M. Y., Litvinchuk A. P. (2018). J. Raman Spectrosc..

[cit26] Cooney R. (1974). Aust. J. Chem..

[cit27] Ammlung R., Shriver D., Kamimoto M., Whitmore D. (1977). J. Solid State Chem..

[cit28] Xu C., Zhang P., Yan L. (2001). J. Raman Spectrosc..

[cit29] Tauc J., Grigorovici R., Vancu A. (1966). Phys. Status Solidi B.

[cit30] Parasyuk O., Khyzhun O., Piasecki M., Kityk I., Lakshminarayana G., Luzhnyi I., Fochuk P., Fedorchuk A., Levkovets S., Yurchenko O. (2017). Mater. Chem. Phys..

[cit31] Yousefi R., Soofivand F., Salavati-Niasari M. (2017). J. Mater. Sci.: Mater. Electron..

[cit32] Rehman S., Mumtaz A., Hasanain S. (2011). J. Nanopart. Res..

[cit33] Hussain M., Ceccarelli R., Marchisio D., Fino D., Russo N., Geobaldo F. (2010). Chem. Eng. J..

[cit34] Sun J., Gao L., Zhang Q. (2003). J. Am. Ceram. Soc..

[cit35] Choina J., Kosslick H., Fischer C., Flechsig G.-U., Frunza L., Schulz A. (2013). Appl. Catal., B.

[cit36] Parida K., Parija S. (2006). Sol. Energy.

[cit37] Low J., Yu J., Jaroniec M., Wageh S., Al-Ghamdi A. A. (2017). Adv. Mater..

[cit38] Malefane M. E. (2020). ACS Omega.

[cit39] Malefane M. E., Feleni U., Kuvarega A. T. (2019). New J. Chem..

[cit40] Jaiswal R., Patel N., Kothari D., Miotello A. (2012). Appl. Catal., B.

[cit41] Hassanpour M., Safardoust-Hojaghan H., Salavati-Niasari M. (2017). J. Mol. Liq..

[cit42] Mafa P. J., Patala R., Mamba B. B., Liu D., Gui J., Kuvarega A. T. (2020). Chem. Eng. J..

[cit43] Schneider J. T., Firak D. S., Ribeiro R. R., Peralta-Zamora P. (2020). Phys. Chem. Chem. Phys..

[cit44] Makama A., Salmiaton A., Choong T., Hamid M., Abdullah N., Saion E. (2020). Chemosphere.

[cit45] Samiee S., Bahmaie M., Motamedi H., Shiralinia A., Gable R. W. (2020). Polyhedron.

[cit46] Kotharkar S. A., Shinde D. B. (2006). Bull. Korean Chem. Soc..

[cit47] Du T., Chen S., Zhang J., Li T., Li P., Liu J., Du X., Wang S. (2020). Nanomaterials.

[cit48] Karami M., Ghanbari M., Alshamsi H. A., Rashki S., Salavati-Niasari M. (2021). Inorg. Chem. Front..

